# Increased Risk of Dementia in Patients Exposed to Nitrogen Dioxide and Carbon Monoxide: A Population-Based Retrospective Cohort Study

**DOI:** 10.1371/journal.pone.0103078

**Published:** 2014-08-12

**Authors:** Kuang-Hsi Chang, Mei-Yin Chang, Chih-Hsin Muo, Trong-Neng Wu, Chiu-Ying Chen, Chia-Hung Kao

**Affiliations:** 1 Department of Public Health, China Medical University, Taichung, Taiwan; 2 Department of Medical Research, Taichung Veterans General Hospital, Taichung, Taiwan; 3 Department of Medical Laboratory Science and Biotechnology, School of Medical and Health Sciences, Fooyin University, Kaohsiung, Taiwan; 4 Management Office for Health Data, China Medical University Hospital, Taichung, Taiwan; 5 Graduate Institute of Clinical Medical Science, College of Medicine, China Medical University, Taiwan; 6 Department of Nuclear Medicine and PET Center, China Medical University Hospital, Taichung, Taiwan; “Mario Negri” Institute for Pharmacological Research, Italy

## Abstract

**Background:**

The air pollution caused by vehicular emissions is associated with cognitive decline. However, the associations between the levels of nitrogen dioxide (NO_2_) and carbon monoxide (CO) exposure and dementia remain poorly defined and have been addressed in only a few previous studies.

**Materials and Methods:**

In this study, we obtained data on 29547 people from the National Health Insurance Research Database (NHIRD) of Taiwan, including data on 1720 patients diagnosed with dementia between 2000 and 2010, and we evaluated the risk of dementia among four levels of air pollutant. Detailed data on daily air pollution were available from January 1, 1998 to December 31, 2010. Yearly average concentrations of pollutants were calculated from the baseline to the date of dementia occurrence, withdrawal of patients, or the end of the study, and these data were categorized into quartiles, with Q1 being the lowest level and Q4 being the highest.

**Results:**

In the case of NO_2_, the adjusted hazard ratios (HRs) of dementia for all participants in Q2, Q3, and Q4 compared to Q1 were 1.10 (95% confidence interval (CI), 0.96–1.26), 1.01 (95% CI, 0.87–1.17), and 1.54 (95% CI, 1.34–1.77), and in the case of CO, the adjusted HRs were 1.07 (95% CI, 0.92–1.25), 1.37 (95% CI, 1.19–1.58), and 1.61 (95% CI, 1.39–1.85).

**Conclusion:**

The results of this large retrospective, population-based study indicate that exposure to NO_2_ and CO is associated with an increased risk of dementia in the Taiwanese population.

## Introduction

Ambient air pollution includes solid and gaseous pollutants [1], [2]. Most of the studies that have investigated the effects of pollutants on cognitive functions have examined the influence of solid pollutants [3]–[8]. However, exposure to ambient gaseous pollutants such as nitrogen dioxide (NO_2_) is known to increase the risk of cerebrovascular and neurodegenerative diseases and ischemic stroke [9]–[12]. Cerebrovascular disease is the principal contributor to dementia [13], [14], and Alzheimer's disease (AD) is the most common neurodegenerative disease. Moreover, a population-base study reported that dementia often developed after the occurrence of an ischemic stroke [15]. Several previous studies have suggested negative associations between NO_2_ exposure and cognitive development in children, including preschool children [16]–[18], and animal studies have indicated that NO_2_ exposure inhibits the recovery of nerve function after a stroke [19], [20]. In addition, one animal study reported that nitration can induce beta-amyloid aggregation and plaque formation [21]; beta-amyloid aggregation is a pathologic hallmark of AD. However, a literature search indicated that only a few studies have been conducted to address the link between NO_2_ exposure and cognitive function in adults. In a recent study conducted on 1496 middle-aged people living in Los Angeles, no statistically significant correlation was detected between the level of NO_2_ exposure and cognitive functions [22]. Therefore, we conducted a retrospective cohort study to determine the association between NO_2_ and dementia risk. Furthermore, in this study, we evaluated the influence of carbon monoxide (CO), because acute CO poisoning may cause headache, nausea, malaise, and fatigue [23], and chronic CO exposure has been linked to depression, confusion, memory loss, and cognitive decline [24], [25]. Comparison between this study with other environmental study of Taiwan NHRID, the main difference is the residential area definition. In previous studies, the residential area is as the insurance area [26]. In present study, we defined the residential areas as the location of clinics which subjects sought treatment for acute upper respiratory infections.

## Materials and Methods

### Data sources and study population

In March 1995, the Taiwan National Health Insurance (NHI) program, which is a single-payer, compulsory social insurance system that has provided insurance coverage to almost every citizen in Taiwan, was established. The NHI covered approximately 99% of the 22.96 million citizens in Taiwan at the end of 2007 [27]. To protect patient privacy, the data on patient identities are encrypted in the National Health Insurance Research Database (NHIRD), and the database is accessible to researchers and the public in Taiwan. In this study, we used a subset of the NHIRD data containing comprehensive health-care data, including files on ambulatory care claims, inpatient claims, and prescriptions received by 1000000 people who were randomly selected from all insured beneficiaries. These data files can be linked through an encrypted but unique personal identification number and, thus, provide a longitudinal medical history of each patient. The health status of each person was identified according to the International Classification of Disease, Ninth Revision, Clinical Modification (ICD-9-CM).

### Exposure assessment

Across Taiwan, 74 ambient air quality monitoring stations are located based on population density. Air quality data are maintained by Taiwan Environmental Protection Administration. [28]. A database containing daily NO_2_ and CO concentrations measured at the monitoring stations was available for the period from January 1, 1998 to December 31, 2010. The people included in this study were assigned pollutant-exposure values based on the data obtained from the monitoring station present in the residential district in which the clinic where the people most frequently sought treatment for acute upper respiratory infection was located (ICD-9-CM Code 460). Yearly average concentrations of pollutants were calculated from the baseline to the date of dementia occurrence, the withdrawal of patients, or the end of the study period, and the data were categorized into quartiles.

### Study patients

We identified 29547 people who were aged 50 years or older and for whom estimable air pollution data were available, but who did not present a history of head injury (ICD-9-CM Codes 800.804, 850.854.1, 310.2, and 959.01), stroke (ICD-9-CM Codes 430–438), or dementia (ICD-9-CM Codes 290.0–290.4, 294.1, and 331.0) before 2000.

### Data Availability Statement

All data and related metadata are deposited in an appropriate public repository: The study population's data were from Taiwan NHIRD (http://w3.nhri.org.tw/nhird//date_01.html) are maintained by Taiwan National Health Research Institutes (http://nhird.nhri.org.tw/) [27]. The National Health Research Institutes (NHRI) is a non-profit foundation established by the government. Air quality data were from Taiwan Air Quality Monitoring Network (http://taqm.epa.gov.tw/taqm/en/PsiMap.aspx) in Taiwan Environmental Protection Administration (http://www.epa.gov.tw/) [28].

### Ethics statement

Because identification numbers of patients had been encrypted, patient consent was not required for this study. This study was approved by the Research Ethic Committee at China Medical University (CMU-REC-101-012). The committee waived the requirement for consent.

### Statistical analysis

We used 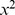 tests to examine the distributions of sex, monthly income (New Taiwan Dollar<14 400, 14 400–18 300, 18 301–21 000, and >21 000), diabetes (DM, ICD-9-CM Code 250), ischemic heart disease (IHD, ICD-9-CM Codes 410–414), hypertension (HT, ICD-9-CM Codes 401–405),chronic obstructive pulmonary disease(COPD, ICD-9-CM Codes 490–496), alcoholism (ICD-9-CM Codes 303.305.0andV113),and the quartiles of NO_2_ concentration (ppb; <6652.3, 6652.3–8349.0, 8349.1–9825.5,>9825.5) and CO concentration (ppm; <196.2, 196.2–241.6, 241.7–296.9, >296.9). A one-way analysis of variance (ANOVA) was performed to compare the age among the quartiles of NO_2_ and CO concentrations. We calculated the incidence density rates of dementia in person-years in each quarter stratified according to sex. The incidence rate ratio (IRR) was estimated using a Poisson regression. Univariate and multivariate Cox proportional hazard regression analyses were performed to calculate the hazard ratios (HRs) and 95% confidence intervals (CIs) of the risk of dementia in association with pollutant levels. Multiple models were tested by controlling for age, sex, monthly income, DM, HT, IHD, COPD, alcoholism, and urbanization. Plots of the Kaplan-Meier analysis were used to determine the probability of people remaining without dementia, and the log-rank test was used to evaluate the differences among quartiles of pollutant concentrations. All analyses were performed using SAS 9.2 software (SAS Institute Inc., Cary, NC, USA), and the Kaplan-Meier survival curve was plotted using the Statistical Package for the Social Sciences (Version 15.1; SPSS Inc, Chicago, IL, USA). All tests were considered statistically significant when two-tailed *P* values were <.05.

## Results

We obtained a total of 29547 and 29537 data on daily NO_2_ and CO exposure, respectively. Dementia was not present at the baseline (2000), and 1720 people developed dementia after follow-up (yearly CO data were available for 1718 people). We categorized the NO_2_ and CO levels into quartiles, with Q1 being the lowest level and Q4 being the highest. The people included in this study had a mean age of 61.4 years (SD 8.5 y). In both the NO_2_ and CO groups, the highest level of the quartiles was associated with the people being slightly younger, more frequently earning a high monthly income, and living in a highly urbanized residential area, but less frequently exhibiting IHD and COPD compared with other quartiles (Tables 1 and 2). Table 3 shows the associations between the gaseous pollutant levels and the risk of dementia. Among the quartiles Q1, Q2, Q3, and Q4 of NO_2_ in all patients, the IRRs in Q2, Q3, and Q4 compared with that in Q1 were 1.05, 0.90, and 1.35, and the adjusted HRs of dementia were 1.10 (95% CI, 0.96–1.26), 1.01 (95% CI, 0.87–1.17), and 1.54 (95% CI, 1.34–1.77), respectively. Among men, we determined that the IRRs in Q2, Q3, and Q4 compared with that in Q1 were 1.08, 0.79, and 1.28, and the adjusted HRs were 1.16 (95% CI, 0.95–1.43), 0.89 (95% CI, 0.71–1.11), and 1.52 (95% CI, 1.23–1.88), respectively. Among women, the IRRs in Q2, Q3, and Q4 compared with that in Q1 were 1.05, 1.11, and 1.56, and the adjusted HRs were 1.05 (95% CI, 0.87–1.27), 1.11 (95% CI, 0.92–1.35), and 1.56 (95% CI, 1.29–1.87), respectively. When the data on sex were stratified or merged for analysis, statistically significant correlations of IRRs and adjusted HRs were measured in Q4 compared with those in Q1.

**Table 1:** Comparison of Baseline Characteristics among quartiles of NO_2_ yearly average.

	Quartiles of NO_2_ yearly average								p	Total (n = 29547)	
	Q1 (n = 7349)		Q2 (n = 7425)		Q3 (n = 7572)		Q4 (n = 7201)				
Dementia	406	5.5	425	5.7	374	4.9	515	7.2	<0.001	1720	5.8
Age (mean, SD)	61.8	8.4	61.4	8.5	61.0	8.4	61.4	8.8	<0.001†	61.4	8.5
Male	3365	45.8	3469	46.7	3474	45.9	3298	45.8	0.611	13606	46.0
Monthly income											
<14400	1481	20.2	1814	24.4	2004	26.5	1991	27.7	<0.001	7290	24.7
14400–18300	1054	14.3	1324	17.8	1511	20.0	1480	20.6		5369	18.2
18301–21000	3255	44.3	2399	32.3	1992	26.3	1785	24.8		9431	31.9
>21000	1559	21.2	1887	25.4	2062	27.2	1944	27.0		7452	25.2
DM	845	11.5	837	11.3	916	12.1	850	11.8	0.421	3448	11.7
IHD	1347	18.3	1354	18.2	1295	17.1	1222	17.0	0.047	5218	17.7
HT	2899	39.4	2906	39.1	2889	38.2	2785	38.7	0.391	11479	38.8
COPD	2612	35.5	2608	35.1	2579	34.1	2376	33.0	0.005	10175	34.4
Alcoholism	19	0.3	19	0.3	22	0.3	10	0.1	0.250	70	0.2
Urbanization											
Highly urbanization	1330	18.1	1668	22.5	2503	33.1	3720	51.7	<0.001	9221	31.2
Moderate urbanization	2157	29.4	2782	37.5	2908	38.4	1828	25.4		9675	32.7
Boomtown	907	12.3	986	13.3	1485	19.6	1126	15.6		4504	15.2
General town	1692	23.0	1160	15.6	412	5.4	298	4.1		3562	12.1
Aging town	304	4.1	56	0.8	68	0.9	72	1.0		500	1.7
Agricultural town	658	9.0	321	4.3	111	1.5	88	1.2		1178	4.0
Remote town	301	4.1	452	6.1	85	1.1	69	1.0		907	3.1

Chi-square test;

† T-test;

**Table 2:** Comparison of Baseline Characteristics among quartiles of CO yearly average.

	Quartiles of CO yearly average								p	Total (n = 29537)	
	Q1 (n = 7565)		Q2 (n = 6428)		Q3 (n = 7681)		Q4 (n = 7863)				
Dementia	391	5.2	321	5.0	476	6.2	530	6.7	<0.001	1718	5.8
Age (mean, SD)	61.8	8.3	61.1	8.3	61.4	8.6	61.3	8.8	<0.001†	61.4	8.5
Male	3532	46.7	2882	44.8	3587	46.7	3597	45.7	0.084	13598	46.0
Monthly income											
<14400	1477	19.5	1477	23.0	2190	28.5	2144	27.3	<0.001	7288	24.7
14400–18300	1074	14.2	1189	18.5	1513	19.7	1591	20.2		5367	18.2
18301–21000	3401	45.0	2095	32.6	1887	24.6	2046	26.0		9429	31.9
>21000	1613	21.3	1667	25.9	2088	27.2	2080	26.5		7448	25.2
DM	862	11.4	712	11.1	918	12.0	954	12.1	0.173	3446	11.7
IHD	1430	18.9	1054	16.4	1394	18.1	1339	17.0	<0.001	5217	17.7
HT	2980	39.4	2455	38.2	3021	39.3	3017	38.4	0.306	11473	38.8
COPD	2785	36.8	2189	34.1	2607	33.9	2587	32.9	<0.001	10168	34.4
Alcoholism	19	0.3	15	0.2	24	0.3	12	0.2	0.232	70	0.2
Urbanization											
Highly urbanization	912	12.1	1697	26.4	2694	35.1	3918	49.8	<0.001	9221	31.2
Moderate urbanization	2388	31.6	2615	40.7	2323	30.2	2346	29.8		9672	32.7
Boomtown	1084	14.3	819	12.7	1576	20.5	1024	13.0		4503	15.2
General town	1684	22.3	772	12.0	781	10.2	322	4.1		3559	12.0
Aging town	336	4.4	22	0.3	65	0.8	74	0.9		497	1.7
Agricultural town	699	9.2	253	3.9	120	1.6	106	1.3		1178	4.0
Remote town	462	6.1	250	3.9	122	1.6	73	0.9		907	3.1

Chi-square test;

† T-test;

**Table 3:** Comparisons of difference dementia incidences and associated hazard ratios among four levels of air pollutants by gender stratification.

			Dementia	PY	Incidence rate#	IRR*	95%CI	aHR†	95%CI
NO_2_	Total	Q1	406	75461.4	5.38	1.00		1.00	
		Q2	425	75246.1	5.65	1.05	0.92, 1.20	1.10	0.96, 1.26
		Q3	374	77576.5	4.82	0.90	0.78, 1.03	1.01	0.87, 1.17
		Q4	515	71461.0	7.21	1.35	1.18, 1.54	1.54	1.34, 1.77
	Male	Q1	186	33853.8	5.49	1.00		1.00	
		Q2	206	34587.2	5.96	1.08	0.89, 1.32	1.16	0.95, 1.43
		Q3	152	34973.3	4.35	0.79	0.64, 0.98	0.89	0.71, 1.11
		Q4	224	31976.0	7.01	1.28	1.05, 1.56	1.52	1.23, 1.88
	Female	Q1	220	41607.6	5.29	1.00		1.00	
		Q2	219	40658.9	5.39	1.02	0.85, 1.23	1.05	0.87, 1.27
		Q3	222	42603.2	5.21	0.99	0.82, 1.19	1.11	0.92, 1.35
		Q4	291	39485.0	7.37	1.41	1.18, 1.67	1.56	1.29, 1.87
CO	Total	Q1	391	77816.4	5.02	1.00		1.00	
		Q2	321	66509.7	4.83	0.96	0.83, 1.11	1.07	0.92, 1.25
		Q3	476	77215.4	6.16	1.23	1.08, 1.41	1.37	1.19, 1.58
		Q4	530	78172.7	6.78	1.36	1.19, 1.55	1.61	1.39, 1.85
	Male	Q1	182	35681.8	5.10	1.00		1.00	
		Q2	145	29334.5	4.94	0.97	0.78, 1.20	1.16	0.93, 1.45
		Q3	212	35371.7	5.99	1.18	0.97, 1.44	1.28	1.04, 1.58
		Q4	227	34977.3	6.49	1.28	1.05, 1.55	1.57	1.26, 1.94
	Female	Q1	209	42134.6	4.96	1.00		1.00	
		Q2	176	37175.2	4.73	0.95	0.78, 1.16	1.01	0.82, 1.24
		Q3	264	41843.8	6.31	1.28	1.07, 1.54	1.46	1.21, 1.77
		Q4	303	43195.4	7.01	1.43	1.20, 1.70	1.64	1.36, 1.98

Incidence rate^#^, per 1,000 person-years;

IRR*, incidence rate ratio;

Adjusted HR^†^ : multiple analysis including age, sex, monthly income, DM, IHD, HT, COPD, alcoholism and urbanization.

**Figure 1:**
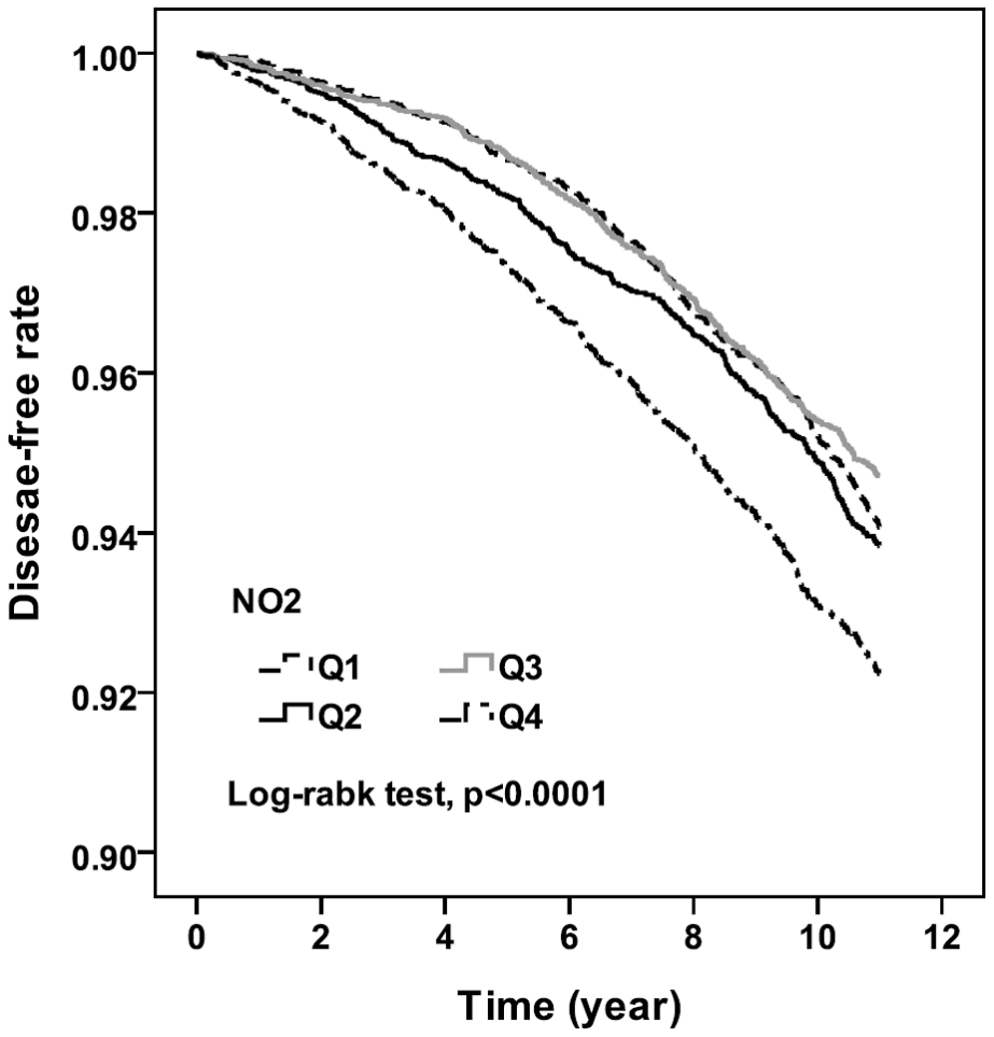
Probability free of dementia among quartiles of yearly average concentration in NO_2_.

**Figure 2:**
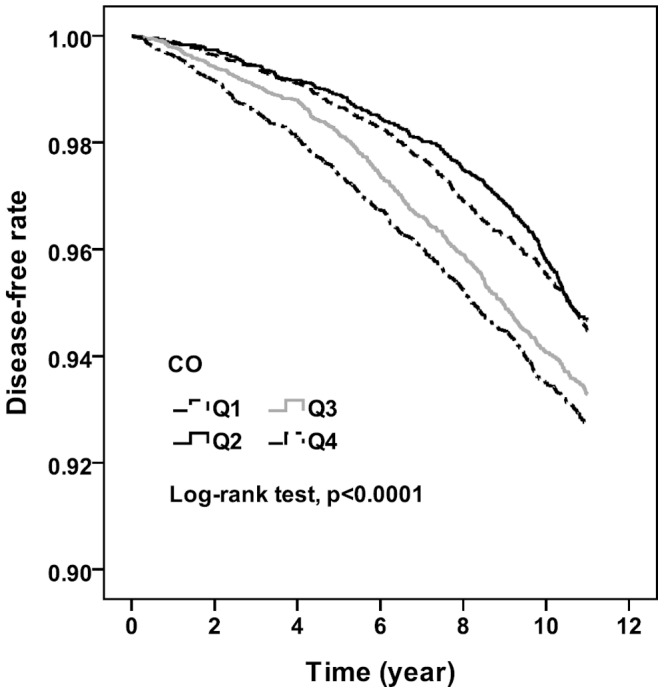
Probability free of dementia among quartiles of yearly average concentration in CO.

Among the quartiles of CO concentration, the IRRs in Q2, Q3, and Q4 compared with that in Q1 were 0.96, 1.23, and 1.36, and the adjusted HRs were 1.07 (95% CI, 0.92–1.25), 1.37 (95% CI,1.19–1.58),and 1.61 (95% CI, 1.39–1.85), respectively, in all people included in the study. Among men, the IRRs in Q2, Q3, and Q4 compared with that in Q1 were 0.97, 1.18, and 1.28, and the adjusted HRs were 1.16 (95% CI, 0.93–1.45), 1.28 (95% CI, 1.04–1.58), and 1.57 (95% CI, 1.26–1.94), respectively. Among women, the IRRs in Q2, Q3, and Q4 compared with that in Q1 were 0.95, 1.28, and 1.43, and the adjusted HRs were 1.01 (95% CI, 0.82–1.24), 1.46 (95% CI, 1.21–1.77), and 1.64 (95% CI, 1.36–1.98), respectively. A clear trend that was detected was an increase in the risk of dementia as CO exposure increased. Figures 1 and 2 show the Kaplan-Meier curves of freedom that were calculated for dementia and are separated according to pollutant level. Statistically significant differences in the occurrence of dementia were observed among the quartiles of NO_2_ and CO concentrations (log-rank test, *P*<.001).

## Discussion

The major finding of previous animal study was that nitration was highly correlated with beta-amyloid aggregation and plaque formation, and beta-amyloid aggregation is a pathologic hallmark of AD [21]. Another animal study indicated that NO_2_ expose can exacerbate the ultra structural impairment of synapses in stroke rats, and induce neuronal damage in healthy rats [29]. The apolipoprotein E (APOE) e4 allele was a well know genetic risk factor or AD, and a randomized clinical trial has found CO poisoning can induce APOE e4 carriers suffer greater morbidity [30].

The major finding of our study was that increased exposure to NO_2_ (Q4) is associated with an enhanced risk of dementia in men and women. The probability of dementia occurrence was increased by 52%–56% in Q4 compared with Q1. A similar trend was observed in the CO group, and the results collectively showed that increasing levels of the 2 pollutants increased the risk of dementia in a dose-dependent manner.

This study was a national population-based investigation on ambient air pollution and dementia. Therefore, collecting individual exposure data was not feasible. To obtain exposure data associated with study patients, previous studies have identified the residential areas of patients by employing a GIS-based system. To protect the privacy of patients, the NHIRD does not provide patients' addresses. Therefore, we identified the residential areas of the patients based on the location of the clinic at which the patients most frequently sought treatment for acute upper respiratory tract infection. In the United States, upper respiratory tract infections are the most common type of infectious disease, and each adult experiences approximately 3 respiratory infections annually [31]. Identifying residential areas in the accessible medical resources, as we did in this study, is more accurate than listing patients according to insurance area [32], [33].

Previous studies have suggested that smoking and drinking alcohol are highly correlated with the risk of AD [34]–[40]. Because of the limitations of the NHIRD, we could not obtain data on the smoking or drinking status of the patients. Therefore, we performed multivariate analysis with COPD and alcoholism adjusted in accordance with previous studies that indicated that smoking is a major causative factor in the development of COPD, and in which alcoholism was diagnosed based on drinking patterns and the attitudes of patients [41]–[43]. In Taiwan, women are not encouraged to smoke or drink alcohol, as reflected in the low prevalence of these behaviors among women (3% and 1%, respectively) [44], [45]. We were able to overcome this limitation by stratifying and adjusting the data according to sex [46].

We adjusted for urbanization in the multivariate analysis. The level of urbanization was determined according to population density (number of people/km^2^), the population ratio of people with a college-level education or higher, the population ratio of people aged over 65 years, the population ratio of agricultural workers, and the number of physicians per 100000 people [47]. The 359 communities in Taiwan were classified into 7groups: highly urbanized area, moderately urbanized area, boomtown, general town, aging town, agricultural town, and remote town. This classification method has been used in several studies [48]–[50].

In addition, we obtained results contrasting those related to dementia, as shown in Tables 1 and 2: the frequency of IHD and COPD were low at the highest level of the pollutants. These results agree with the explanation provided by previous studies suggesting that patients who are highly educated and earn a high monthly income live in areas where the level of air pollutants is high [6], [22].

The strengths of this study are the following. First, this study was based on a long follow-up period, which allowed the possible occurrence of dementia to be assessed. Second, Taiwan launched a national health insurance (NHI) in 1995, operated by a single-buyer, the government. All insurance claims should be scrutinized by medical reimbursement specialists and peer review. The diagnoses of dementia were based on the ICD-9 code determined by qualified clinical neurology physicians under strict audit in the reimbursement process. Therefore the diagnoses and codes for dementia should be accurate and reliable. Third, this study was conducted using a large population derived from the NHIRD. In Taiwan, the government is the only compulsory social insurance provider; approximately 99% of the 23.74 million citizens of Taiwan are enrolled in the NHI program. Because this was a nationwide study, we considered urbanized towns throughout Taiwan. Lastly, in this study, cerebrovascular and cardiovascular diseases were considered and the association between pollutants and dementia was evaluated. We excluded subjects with cardiovascular before the index date in this study because cardiovascular was a widely known predictor for dementia. IHD increased 27% risk for dementia in both model 1 and model 2. (Table S1).

Certain limitations of this study should be considered. First, the evidence derived from a retrospective cohort study is generally lower in statistical quality than that obtained from randomized trials because, in such retrospective studies, potential biases exist that are related to the adjustment of confounding variables. Despite our meticulous study design and the measures adopted to control for confounding factors, bias resulting from unknown confounders may have affected our results. Second, all data in the NHIRD are anonymous. Thus, relevant clinical variables, such as imaging results and pathology findings, were unavailable for the patient cases included in this study. Third, the participants were assigned to residential districts based on the clinic where they most frequently sought treatment for acute upper respiratory infection. Therefore, the resident who has no acute upper respiratory infection during study period had being excluded in this study. In our opinion, the resident without respiratory infection related medical record exposed to low level air pollutants. It might under the estimated risk of dementia. Nevertheless, the data on air pollutants and dementia diagnoses were reliable.

### Conclusions

Understanding the regional distribution of human health statuses can facilitate the investigation of the spread of diseases and the related risk factors as well as the assessment of medical resources and the planning of the use of these resources. In future research, animal studies can be conducted to further examine the association between air pollutants and neurological disorders.

## Supporting Information

Table S1 **Adjusted hazard ratio for dementia and dementia-associated risk factors.**
(DOCX)
