# Supplementary material for: Increased Risk of Dementia in Patients Exposed to Nitrogen Dioxide and Carbon Monoxide: A Population-Based Retrospective Cohort Study
Source: PLoS One. 2014 Aug 12;9(8):e103078. doi: 10.1371/journal.pone.0103078 (PMC4130523; doi:10.1371/journal.pone.0103078)
Supplement: Table S1 — Adjusted hazard ratio for dementia and dementia-associated risk factors. (DOCX) [file pone.0103078.s001.docx]

|  | Supplementary Table 1. Adjusted hazard ratio for dementia and dementia-associated risk factors | | | | | |
| --- | --- | --- | --- | --- | --- | --- |
| HR (95%CI) | | | Model 1 | | Model 2 | |
| Age | |  | 1.12 | (1.11, 1.12) | 1.12 | (1.11, 1.12) |
| Male vs. female | |  | 0.85 | (0.77, 0.94) | 0.86 | (0.78, 0.95) |
| Monthly income | | <14400 | 1.00 |  | 1.00 |  |
|  | | 14400-18300 | 1.03 | (0.75, 1.42) | 1.03 | (0.75, 1.43) |
|  | | 18301-21000 | 0.84 | (0.61, 1.16) | 0.84 | (0.61, 1.15) |
|  | | >21000 | 0.92 | (0.66, 1.29) | 0.93 | (0.66, 1.30) |
| DM | |  | 1.35 | (1.18, 1.53) | 1.36 | (1.19, 1.54) |
| IHD | |  | 1.27 | (1.13, 1.42) | 1.27 | (1.13, 1.42) |
| HT | |  | 1.03 | (0.93, 1.14) | 1.04 | (0.93, 1.15) |
| COPD | |  | 1.15 | (1.04, 1.27) | 1.15 | (1.04, 1.27) |
| Alcoholism | |  | 3.15 | (1.50, 6.62) | 3.12 | (1.48, 6.55) |
| Urbanization | | Highly urbanization | 1.00 |  | 1.00 |  |
|  | | Moderate urbanization | 1.18 | (1.04, 1.33) | 1.18 | (1.04, 1.33) |
|  | | Boomtown | 1.07 | (0.91, 1.25) | 1.05 | (0.90, 1.23) |
|  | | General town | 1.35 | (1.14, 1.60) | 1.31 | (1.11, 1.55) |
|  | | Aging town | 1.35 | (0.96, 1.90) | 1.29 | (0.92, 1.81) |
|  | | Agricultural town | 1.48 | (1.16, 1.88) | 1.39 | (1.10, 1.77) |
|  | | Remote town | 1.53 | (1.16, 2.02) | 1.43 | (1.08, 1.88) |
| NO_2_ | | Q1 | -- |  | 1.00 |  |
|  | | Q2 | -- |  | 1.10 | (0.96, 1.26) |
|  | | Q3 | -- |  | 1.01 | (0.87, 1.17) |
|  | | Q4 | -- |  | 1.54 | (1.34, 1.77) |
| CO | | Q1 | 1.00 |  | -- |  |
|  | | Q2 | 1.07 | (0.92, 1.25) | -- |  |
|  | | Q3 | 1.37 | (1.19, 1.58) | -- |  |
|  | | Q4 | 1.61 | (1.39, 1.85) | -- |  |
